# Supplementary material for: Immunoinformatics design of a novel multiepitope vaccine candidate against non-typhoidal salmonellosis caused by Salmonella Kentucky using outer membrane proteins A, C, and F
Source: PLoS One. 2025 Jan 10;20(1):e0306200. doi: 10.1371/journal.pone.0306200 (PMC11723559; doi:10.1371/journal.pone.0306200)
Supplement: Attachment — Submitted filename: PONE-D-24-23783_Review_Ruchir Pansuriya.docx [file pone.0306200.s001.docx]

**PONE-D-24-23783**

**General comment:**

The research article "Immunoinformatics design of a novel multiepitope vaccine candidate against non-typhoidal salmonellosis caused by *Salmonella Kentucky* using outer membrane proteins A, C, and F". The research demonstrates a well-structured approach to vaccine design using bioinformatics and immunoinformatics techniques. The study created a novel MEV construct from OMP A, C, and F with the inclusion of fliC protein as an adjuvant from Salmonella typhimurium and characterized it using various software. The MEV was identified as having 14 CD8+ and 7 CD4+ T-cell epitopes, along with immunogenic B-cell epitopes. The vaccine construct demonstrated high structural quality, favorable physicochemical properties, and stability in molecular docking and dynamic simulations. Immune simulations indicated significant dose-dependent immune responses of IgG and IgM. The MEV is predicted to be non-toxigenic and non-reactogenic in nature.

**Specific comments:**

The authors used immunoinformatics tools to construct a potential MEV candidate for *Salmonella Kentucky* and characterized it with various software / in-silico tools to predict interferon-gamma (IFN-γ) 420 induction capabilities, discontinuous B-cell epitopes, secondary and tertiary structure, Protein-protein docking and protein-protein binding affinity, Molecular dynamics, and Immune Simulation. The authors also cited various reference (38, 47, 48, 54, 62, 63, 66, 73, 77, 78, 81, 82) which has used similar approach of immunoinformatics to design vaccine for various infectious diseases. However, the finding does not provide experimental data to support the theoretical findings, which is crucial for translating the research into a viable vaccine candidate. This study could benefit from a more detailed discussion of the potential challenges and limitations of this approach. Additionally, it is important to cite the reference who used the immunoinformatics approach to design a vaccine candidate and evaluated it in small animals to support in-silico Vs in-vivo results.

**Section 2.8:**

- Line no 320 - “Ensuring the efficacy of the vaccine model during cloning and expression is crucial for its in-vitro production” should be changed to “Ensuring the efficacy of the vaccine model during cloning and expression is crucial for its in-vivo production”
- It is not clear why authors in this study performed codon optimization for a vaccine construct for a mammalian host. The MEV proposed is a bacterial-derived chimeric protein construct that can be easily expressed in a simple host like *E. coli.* This poses a question on the rationale for the construction of MEV codon.

**Section 2.19:**

- It is not clear why the authors introduced 6xHis tag on the C-terminal of the MEV in this study as all the experiments were in silico. It is recommended to avoid His tags in vaccine candidate when predicting their immune response using the database. The MEV containing His tag may potentially lead to the generation of anti-histamine antibodies.
- Authors have also not described whether any antibiotic marker is used in a plasmid construct.

**Section 3.6 and discussion section:**

The authors have not described the rationale for the order of linkage B cell, HTL and CTL epitope peptide linkage in a MEV construct. Was it random or a particular order of different peptide sequences of epitopes were followed to construct MEV?

**Section 3.12 and discussion section:**

- The immune simulation predicted the humoral responses 565 for both IgM and IgG were more pronounced after booster doses compared to the initial dose, with IgM levels being higher than IgG (and its isotypes). A detailed discussion would be beneficial to describe the significance of a higher level of IgM as compared to IgG and what it means.
- A detailed discussion on the rationale of three doses and dose intervals while performing an immune stimulation.

**Section 5.0:**

Line no. 732: The author claimed MEV to be thermostable. However, the rationale for this claim is neither mentioned in the result section nor described in the discussion section. What parameters or scores were responsible for predicting good solubility and thermos-stability?
