# Supplementary material for: Immunoinformatics design of a novel multiepitope vaccine candidate against non-typhoidal salmonellosis caused by Salmonella Kentucky using outer membrane proteins A, C, and F
Source: PLoS One. 2025 Jan 10;20(1):e0306200. doi: 10.1371/journal.pone.0306200 (PMC11723559; doi:10.1371/journal.pone.0306200)
Supplement: Attachment — Submitted filename: Response to Reviewers.docx [file pone.0306200.s002.docx]

**PONE-D-24-23783**

**Key areas addressed as requested by the academic editor**

1. To better understand the antigenic diversity for the different classes of OMPs (OmpA, OmpC, and OmpF), the percentage conservation of the amino acid sequence has been computed using the Phylogeny.fr server (Section 2.2 line 121), the results are in section 3.1 and table 2. The phylogenetic relationships, based on the maximum likelihood method, have been computed as recommended (Fig 2).
2. The language and structure have been greatly improved as recommended. Several paragraphs have been restructured to enhance clarity, professional tone, and flow. Attention has been given to punctuation details, abbreviations, table headers, and legends.
3. A flow chart explaining the methods in a stepwise manner has been included in the manuscript as recommended (Fig 1). The methodologies were improved upon, and the rationale behind the methods used was stated. You can find them in sections 2.2 (Lines 121 to 129 ), 2.9 (lines 223 to 226), section 2.17 (Lines 333 to 344), section 2.18 (lines 349 to 354 ), and 2.19 (lines 376 to 383)
4. The potential challenges and limitations of the use of the immunoinformatics approach have been provided (Section 6.0). Finally, I have incorporated references to studies that have used an *in-silico* approach to vaccine design with *in-vivo* evaluations (References 18, 35, 47, 69, 70, and 71)

**Response to reviewers**

Response to Reviewer # 1

1. Outer membrane proteins (OMPs) are highly conserved and identical within each class of protein in *Salmonella*, regardless of the serovar (as stated in lines 80 to 83)*.* However, the percentage conservation across different classes of amino acid sequences has been computed and presented in section 3.1 (lines 394 to 403) and Table 2.

To avoid ambiguity, section 2.1 has been rephrased to clarify intent. The reason for blasting at 99 % to 100% for each class of OMP in our study was majorly to narrow down our selection criteria, to ensure all selected proteins were those isolated from *Salmonella* Kentucky. This does not in any way narrow the antigenic range within each class of protein.

2. To better understand the antigenic diversity for the different classes of OMPs (OmpA, OmpC, and OmpF), a phylogenetic tree based on maximum likelihood has been computed and added as recommended (Fig 2).

Response to Reviewer # 2

1. Punctuations have been improved, typographical errors corrected and the right terminologies noted.
2. A statement on the various risk factors that make Africa prone to the emergence of AMR clones has been added (Lines 90 to 93).
3. All species names have been italicized
4. The IC50 threshold value chosen has been stated (Line 160).
5. Tables have been updated to differentiate epitopes by protein classes (Tables 3 and 6).
6. The use of “amino acid” in FASTA format has been clearly stated (Section 2.1)
7. Legends for figures 3, 4, 5, 6, and 12–16, have been updated to improve clarity and conciseness.
8. Figure 9, formerly Figure 7, depicting the dock complex between TLRs and MEV, has been updated to include the color coding between MEV and TLRs, as recommended.

Response to Reviewer # 3

1. References to studies that used the immunoinformatics approach to design a vaccine candidate and evaluated it in small animals to support *in-silico* Vs *in-vivo* results have been included (18, 35, 47, 69, 70, 71).
2. The rationale for the choice of plasmid and the choice of dosing regimen has been explained in the manuscript as advised and references that have used similar approaches are cited (Section 2.17, Line 333–344; Section 2.18, line 349–355). References 39, 65, 66, 67, 68.
3. Codon optimization was performed for mammalian hosts because the vaccine was designed for humans. In vitro experiments (e.g., transfection studies) are often conducted in mammalian cell lines for human vaccines. Additionally, most in vivo experiments are performed in laboratory animals like mice, rats, and monkeys, which are mammals. Studies that employed mammalian codon optimization and or, the pcDNA3.1 plasmid, have been referenced in the manuscript (sections 2.18 and 2.19), specifically in references 18, 35, 68, 69, and 70.
3. The immune simulation study was done using the amino acid (AA) sequence of the multi-epitope vaccine construct without the 6xHis tag AA sequence. The inclusion of the 6xHis tag was during cloning into the plasmid after the immune simulation had been conducted. Studies with similar approaches have been referenced (Reference 47). The addition of the 6xhis tag to the protein before cloning was to make it easy to track the protein during expression and purification processes when conducting *in-vitro* experiments.

The detection of anti-His antibodies is what makes it easier to track the proteins of interest during expression and purification processes. Finally, 6xHis tag usually does not interfere with the protein’s native structure or function.

4. Most *in-silico* designs employ plasmid with antibiotic markers. They are utilized to identify and successfully select transformants, particularly in cloning, gene expression, and genetic engineering. This study and other studies referenced in this manuscript employed plasmids that carry antibiotic markers. The *AmpR* (ampicillin-resistant) gene carried on our plasmid was the marker (Fig 18).
5. The rationale for the order of linkage of the epitopes for the construction of the MEV has been included in the manuscript (Section 2.9, lines 223–227).
6. A detailed discussion on the implication of the high IgM has been included in the manuscript (Lines 762 – 773).
7. The physicochemical properties of the vaccine that make it thermostable and soluble are available in the results (Section 3.7, Lines 488 to 490 for solubility; and lines 491 to 494 for aliphatic index). These parameters and scores have been quoted in the discussion (lines 735– 739).
