# Supplementary material for: Immunoinformatics design of a novel multiepitope vaccine candidate against non-typhoidal salmonellosis caused by Salmonella Kentucky using outer membrane proteins A, C, and F
Source: PLoS One. 2025 Jan 10;20(1):e0306200. doi: 10.1371/journal.pone.0306200 (PMC11723559; doi:10.1371/journal.pone.0306200)
Supplement: Attachment — Submitted filename: PONE-D-24-23783_Review_2-Ruchir Pansuriya.docx [file pone.0306200.s003.docx]

**PONE-D-24-23783**

**General Comment:**
The authors have addressed most of the comments raised during the first review. The article now includes more comprehensive data, and the addition of much-needed discussion in the relevant sections has strengthened the manuscript.

**Specific Comment (2.18 Codon Adaptation):**
The authors propose using mammalian cell lines for the production of the MEV protein construct. However, approximately half of all proteins expressed in mammalian cells undergo post-translational modifications, such as glycosylation of specific amino acids, which are undesirable when expressing bacterial protein constructs. Have the authors employed any genetic tools to prevent glycosylation of the MEV construct? The use of the enzyme PNGase F (N-Glycosidase F) is an effective method for removing most N-linked oligosaccharides from glycoproteins. It would be helpful to clarify this aspect in sections 3.13 and 4.0. A discussion on this point would provide greater transparency regarding the in-silico work performed.
