# Supplementary material for: Immunoinformatics design of a novel multiepitope vaccine candidate against non-typhoidal salmonellosis caused by Salmonella Kentucky using outer membrane proteins A, C, and F
Source: PLoS One. 2025 Jan 10;20(1):e0306200. doi: 10.1371/journal.pone.0306200 (PMC11723559; doi:10.1371/journal.pone.0306200)
Supplement: Attachment — Submitted filename: Response to Reviewers.docx [file pone.0306200.s004.docx]

**PONE-D-24-23783R1**

**Key areas addressed as requested by Journal**

I have carefully reviewed all references cited in this manuscript to confirm their accuracy and completeness and can confirm that no retracted articles have been cited or identified.

**Response to reviewers**

Response to Reviewer # 1

Yes. The developed vaccine candidate is expected to be effective against all strains of Salmonella Kentucky, including those with and without available sequences in the NCBI database, as the outer membrane protein sequences used in this study incorporate highly conserved regions.

Response to Reviewer # 3

**Response to Specific Comment (2.18 Codon Adaptation):**

The authors appreciate the insightful feedback regarding glycosylation concerns for the MEV construct expressed in mammalian cells.

The authors recognize the potential for glycosylation at certain amino acid residues. Although the in-silico modeling did not integrate genetic interventions to address glycosylation, this issue can be effectively addressed during in-vitro experimentation:

1. **Post-Expression Enzymatic Treatment**: In-vitro assays can utilize PNGase F (N-Glycosidase F) to remove any N-linked glycans after expression enzymatically. While this does not inhibit glycosylation during protein synthesis, it ensures that the final product remains glycosylation-free, making it a closer structural analog to the bacterial protein.
2. **Use of Glycosylation-Deficient Cell Lines:** Certain cell lines have been genetically modified to lack glycosylation pathways, such as CHO-K1 cell lines, which we intend to select for this purpose. Using these glycosylation-deficient cell lines minimizes or eliminates unintended glycosylation during protein expression.

Given that this manuscript focuses exclusively on in-silico findings, the authors have not detailed the use of CHO-K1 cell lines in this research phase. We anticipate that these in-vitro techniques will adequately address glycosylation concerns in future experimentation phases.
